# Antimicrobial, Antibiofilm, and Anticancer Activities of *Syzygium aromaticum* Essential Oil Nanoemulsion

**DOI:** 10.3390/molecules28155812

**Published:** 2023-08-01

**Authors:** Amr M. Shehabeldine, Ahmed S. Doghish, Walaa A. El-Dakroury, Mahmoud M. H. Hassanin, Abdulaziz A. Al-Askar, Hamada AbdElgawad, Amr H. Hashem

**Affiliations:** 1 Botany and Microbiology Department, Faculty of Science, Al-Azhar University, Nasr City 11884, Egypt; 2 Department of Biochemistry, Faculty of Pharmacy, Badr University in Cairo (BUC), Badr City 11829, Egypt; 3 Biochemistry and Molecular Biology Department, Faculty of Pharmacy (Boys), Al-Azhar University, Nasr City 11231, Egypt; 4 Department of Pharmaceutics and Industrial Pharmacy, Faculty of Pharmacy, Badr University in Cairo (BUC), Badr City 11829, Egypt; walaa.ahmed2@buc.edu.eg; 5 Ornamental, Medicinal and Aromatic Plant Disease Department, Plant Pathology Research Institute, Agricultural Research Center (ARC), Giza 12619, Egypt; dr.hassanin.1978@gmail.com; 6 Department of Botany and Microbiology, Faculty of Science, King Saud University, P.O. Box 2455, Riyadh 11451, Saudi Arabia; aalaskara@ksu.edu.sa; 7 Integrated Molecular Plant Physiology Research (IMPRES), Department of Biology, University of Antwerp, 2022 Antwerp, Belgium; hamada.abdelgawad@uantwerpen.be

**Keywords:** clove essential oil, CLSM, nanoemulsion, antimicrobial activity, antibiofilm activity, anticancer activity, apoptosis

## Abstract

In the current study, clove oil nanoemulsion (CL-nanoemulsion) and emulsion (CL-emulsion) were prepared through an ecofriendly method. The prepared CL-nanoemulsion and CL-emulsion were characterized using dynamic light scattering (DLS) and a transmission electron microscope (TEM), where results illustrated that CL-nanoemulsion droplets were approximately 32.67 nm in size and spherical in shape, while CL-nanoemulsion droplets were approximately 225.8 nm with a spherical shape. The antibacterial activity of CL-nanoemulsion and CL-emulsion was carried out using a microbroth dilution method. Results revealed that the preferred CL-nanoemulsion had minimal MIC values between 0.31 and 5 mg/mL. The antibiofilm efficacy of CL-nanoemulsion against *S. aureus* significantly decreased the development of biofilm compared with CL-emulsion. Furthermore, results illustrated that CL-nanoemulsion showed antifungal activity significantly higher than CL-emulsion. Moreover, the prepared CL-nanoemulsion exhibited outstanding antifungal efficiency toward *Candida albicans*, *Cryptococcus neoformans*, *Aspergillus brasiliensis*, *A. flavus*, and *A. fumigatus* where MICs were 12.5, 3.12, 0.78, 1.56, and 1.56 mg/mL, respectively. Additionally, the prepared CL-nanoemulsion was analyzed for its antineoplastic effects through a modified MTT assay for evaluating apoptotic and cytotoxic effects using HepG2 and MCF-7 cell lines. MCF-7 breast cancer cells showed the lowest IC_50_ values (3.4-fold) in CL-nanoemulsion relative to that of CL-emulsion. Thus, CL-nanoemulsion induces apoptosis in breast cancer cells by inducing caspase-8 and -9 activity and suppressing VEGFR-2. In conclusion, the prepared CL-nanoemulsion had antibacterial, antifungal, and antibiofilm as well as anticancer properties, which can be used in different biomedical applications after extensive studies in vivo.

## 1. Introduction

Multidrug-resistant (MDR) bacteria are more common, which highlights a rising concern about appropriate therapies for diseases brought on by these infections [1]. Humans exposed to bacteria that are highly and/or multidrug-resistant have experienced significant cases of illness and mortality due to the absence of appropriate treatments [2]. As a result, microbial resistance is an important issue for the community, and there is a need to research and identify novel chemicals with antimicrobial activities that have no adverse effects on the host body.

Nanotechnology has a wide range of applications across various fields such as medicine, agriculture, electronics, energy, remediation, and water treatment [3,4,5,6,7,8,9,10]. Essential oil nanoemulsion is a type of nanotechnology-based delivery system that allows for the efficient encapsulation and delivery of essential oils. Nanoemulsions are stable, transparent, and homogeneous mixtures of oil, water, and emulsifiers, with droplet sizes typically ranging from 10 to 200 nm [11]. Nanoemulsion enhances the surface area of essential oil by lowering the droplet size, making it easier for the body to absorb and consume [12]. Essential oil nanoemulsion has several possibilities in the food, cosmetics, and medical sectors. They increase flavor and nutritional value in lotions, creams, sprays, and food products [13,14,15,16]. Antimicrobial activity has been observed in essential oil nanoemulsion against a broad spectrum of microorganisms, including bacteria, fungi, and viruses [17,18]. This antimicrobial activity is due to the presence of bioactive compounds in essential oils, which can disrupt the cell membrane of microorganisms and inhibit their growth [19].

Clove is also known as the expanding buds of *Syzygium aromaticum* (L.). Syzygium is the biggest class in the Myrtaceae family, with over 1200–1800 blooming plant varieties [20]. Steam distillation, cold pressing, or supercritical CO_2_ extraction and solvent extraction are methods for obtaining clove essential oils, which are exceptionally concentrated herbal extracts. [21]. Steam distillation is a technique used to separate volatile compounds from nonvolatile substances, typically employed in the extraction of essential oils from plants. Steam distillation is carried out by passing dry steam through the plant material whereby the steam volatile compounds are volatilized, condensed, and collected in receivers [22]. Also, the extraction of oil using supercritical CO_2_ is a popular method in the extraction of oils due to its efficiency, selectivity, and environmentally friendly nature [23]. Supercritical CO_2_ refers to a state of CO_2_ where it is above its critical temperature (31.1 °C) and critical pressure (73.8 bar) [24]. In this state, CO_2_ exhibits unique properties that make it useful in the extraction of oils. Clove essential oil contains over 30 distinct substances, of which eugenol makes up at least 50%, and the other 10–40% is composed of eugenyl acetate, humulene, and caryophyllene [25]. Clove essential oil has been used to heal burns and wounds, as well as anesthesia in dentistry. Furthermore, its usage in many commercial uses has been described, and it is widely employed in fragrances, detergents, and as a washing medium in histology work. Multiple investigations have found that aromatic plants such as clove, thyme, and mint have antibacterial, antiviral, anticarcinogenic, and antifungal properties. However, due to its significant antibacterial and antioxidant properties, clove has earned a lot of popularity among various spices [26]. The advantages associated with plant compounds have been known since time immemorial. However, their advantages are becoming more well understood, owing to their diverse medicinal characteristics [27]. The antibacterial capacity of clove oil nanoemulsion has been discovered to be significantly greater than that of conventional preparation [28].

Since nanotechnology has proven to be an effective technique for treating cancer, researchers have concentrated their efforts on treating a variety of cancers. The effects of CL-nanoemulsion on apoptosis in both breast (MCF-7) and liver (HepG2) cancer cells were evaluated. These promising results suggest that a natural product, CL-nanoemulsion, may hold the key to facilitating the development of cancer treatments, notably for breast cancer. This work aims to prepare CL-nanoemulsion through an ecofriendly method and to characterize it using TEM and DLS, as well as to assess its antibacterial, antibiofilm, antifungal, and anticancer potentialities.

## 2. Results and Discussion

### 2.1. Preparation and Characterization of CL-Nanoemulsion

Clove oil was extracted from *Syzygium aromaticum* using the ecofriendly method. To convert clove oil to emulsion or nanoemulsion, an emulsifying agent must be used through an emulsification process [29]. Emulsification is a process that involves the mixing and stabilization of two immiscible liquids, typically oil and water, to form a stable emulsion. An emulsion is a mixture of tiny droplets of one liquid dispersed in another liquid. Emulsions can be either oil-in-water (O/W) or water-in-oil (W/O), depending on the continuous phase [30]. Thus, in the current study, Tween 80 was used as an emulsifying agent in emulsion and nanoemulsion formation from clove oil. Tween 80 is widely used for the formation of emulsions due to Tween having a high hydrophilic–lipophilic balance (HLB) value, which supports the creation of oil-in-water emulsions [31]. The result revealed that changing color to white indicates the formation of emulsion or nanoemulsion according to the method used. To confirm the formation of CL-nanoemulsion, dynamic light scattering and TEM analyses were carried out.

### 2.2. Dynamic Light Scattering

**Figure 1:**
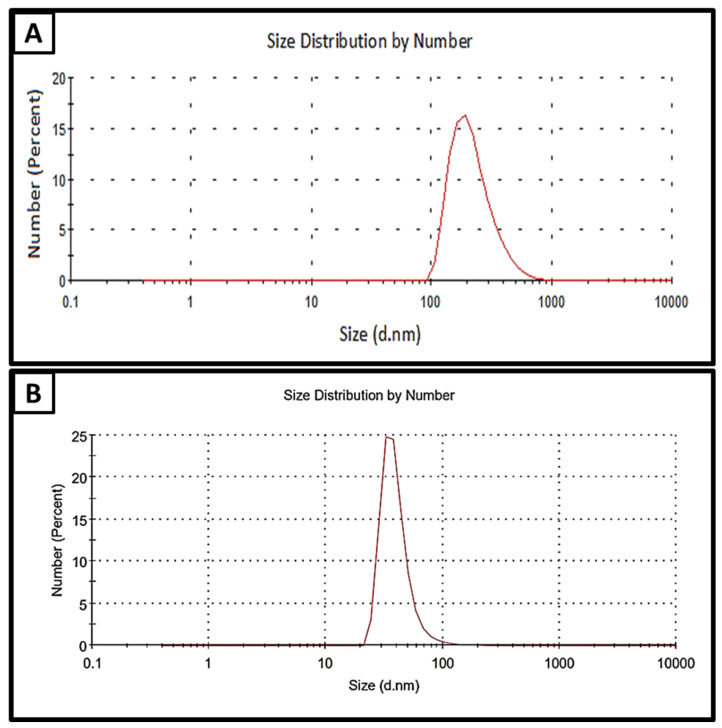
DLS of CL-emulsion (**A**) (peak at 225.8 nm and PDI = 0.242) and CL-nanoemulsion (**B**) (peak at 32.67 nm and PDI = 0.355).

Figure 1 shows the stable CL-nanoemulsion prepared by the ultrasonication method for 40 min at 350 W after 40 days of storage at room temperature. Previous studies confirmed that surfactant concentrations affected significantly the hydrodynamic diameter and polydispersity of nanoemulsions [32,33]. Results revealed that CL-nanoemulsion droplets were approximately 32.67 nm in size; the polydispersity index (PDI) for particles was 0.355, as shown in Figure 1B. On the other hand, CL-emulsion droplets were approximately 225.8 nm in size; the PDI for particles was 0.242, as illustrated in Figure 1A. Dai et al. [34] reported that nanoemulsion has a small droplet size in the presence of double bonds in the nonpolar chain of non-ionic surfactants. Hashem, Abdelaziz, Hassanin, Al-Askar, AbdElgawad, and Attia [18] succeeded in the preparation of CL-nanoemulsion where mean droplets were 91.3 nm and PDI was 0.448. The mean hydrodynamic diameter rose in direct proportion to the quantity of additional clove essential oil because of an increase in the internal capacity of the nanoparticles covered by the oil. This may also be a result of changes in the organic viscosity and physicochemical properties of solvents released into water. Krishnamoorthy et al. [35] prepared cleome viscosa oil nanoemulsion and noticed the dimensions of droplets NE retention at ambient temperature varied significantly, ranging from 10 to 19 nm, 23 to 24 nm, and 163 to 63 nm for 1:3, 1:2, and 1:1 (oil: surfactant (*v*/*v*)), respectively. Enayatifard et al. [36] illustrated that oregano nanoemulsion exhibited low PDI (0.11), and the mean droplet was 72.26 nm.

### 2.3. Transmission Electron Microscopy (TEM)

**Figure 2:**
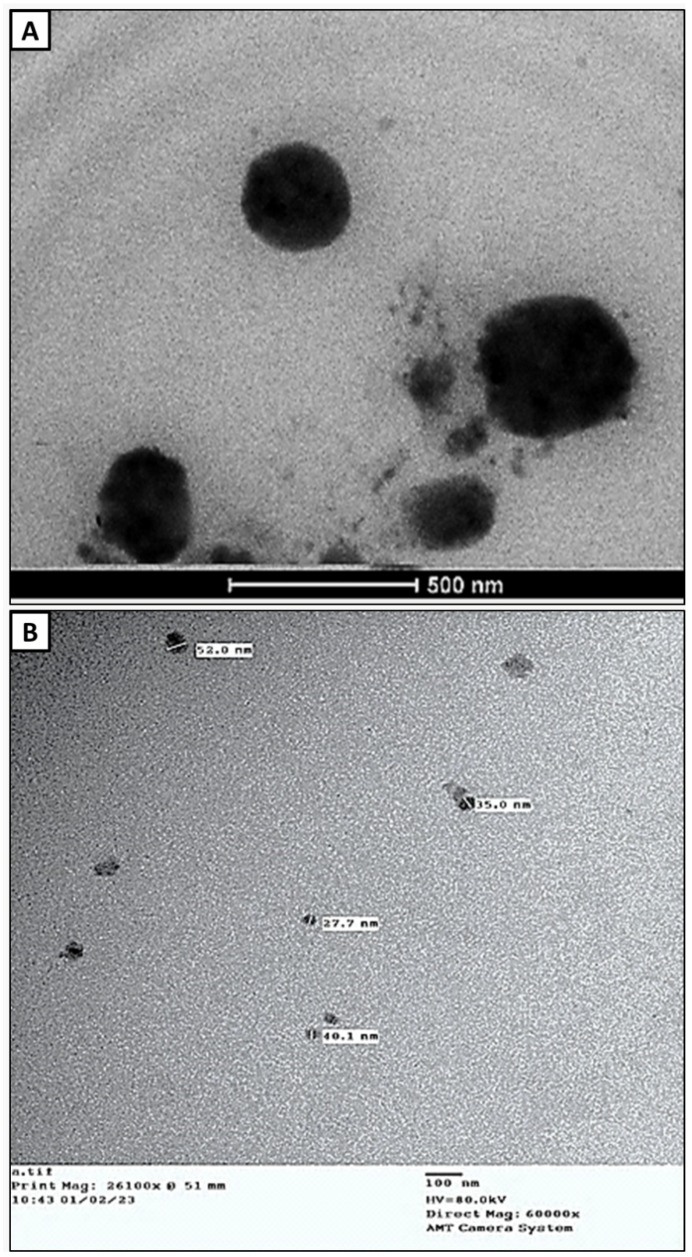
Transmission electron microscopic image of CL-emulsion (**A**) and CL-nanoemulsion (**B**).

The actual dimensions and shape of the CL-nanoemulsion drops are revealed by TEM analysis. The TEM micrograph showed that the CL-nanoemulsion was spherical in shape. CL-nanoemulsion droplets were in the range of 27.7–52 nm (Figure 2B). CL-emulsion was also spherical in shape. CL-emulsion droplets were in the range of 242.9–428.6 nm (Figure 2A). The obtained results were in agreement with Hashem, Abdelaziz, Hassanin, Al-Askar, AbdElgawad, and Attia [18], who found that TEM micrograph CL-nanoemulsion was spherical in shape and droplet size was in the range of 36.4–57.1 nm. Hammad and Hasanin [37] reported that the shape of spearmint and thyme nanoemulsions was spherical with mono- or di-dispersed; also, the size was in the ranges (5.91–9.77) and (25.4–32.9), respectively. Abd-Elsalam and Khokhlov [38] illustrated that TEM results of eugenol oil nanoemulsion appeared spherical and the size was in the range of 50–110 nm.

### 2.4. Determination of MIC and MBC

CL-emulsion and CL-nanoemulsion have good antibacterial activity against both Gram-positive and Gram-negative bacteria, according to extensive research in the literature [39]. The preliminary detection of CL-emulsion and CL-nanoemulsion against tested bacteria was carried out using a microbroth double dilution assay. Using a resazurin-mediated microtiter plate test, a visual evaluation of the inhibitory impact of the test compounds was done using the color shift of the resazurin indicator [40]. MIC of the CL-emulsion against *B. cereus*, *S. aureus*, *E. coli*, and *K. oxytoca* was 1.25, 2.5, 10, and 5 mg/mL, respectively, while MIC of the clove oil nanoemulsion against *B. cereus*, *S. aureus*, *E. coli*, and *K. oxytoca* was 0.31, 0.62, 1.25, and 5 mg/mL, respectively. Our results showed that clove oil nanoemulsion possesses the lowest MIC ranging from 0.31 to 5 mg/mL (Table 1). The MBC is the lowest quantity of CL-nanoemulsion necessary to totally eliminate the bacteria under certain circumstances (no development on the agar dish) [2,41]. MBC of the clove oil nanoemulsion against *B. cereus*, *S. aureus*, *E. coli*, and *K. oxytoca* were 0.62, 1.25, 2.5, and 10 mg/mL, respectively, whereas the MBC of the CL-emulsion was 2.5, 5, 20, and 10 mg/mL, respectively. One of the previous studies by Sharma [42] reported that Gram-negative bacteria such as *E. coli* were shown to be more vulnerable to CL-nanoemulsion than Gram-positive bacteria; this is in agreement with our results. One probable reason is that the walls of their cells differ in content depending on whether they are Gram-positive or Gram-negative. *E. coli* has a distinctive cell membrane with a periplasmic gap that renders it more susceptible to antimicrobial effects [43]. Also, the improvement in CL-emulsion bioavailability within the nanoemulsion leads to homogeneous oil dispersion and discharge that is adequate to suppress bacterial growth.

### 2.5. Antibiofilm Activity of CL-Emulsion and CL-Nanoemulsion

**Figure 3:**
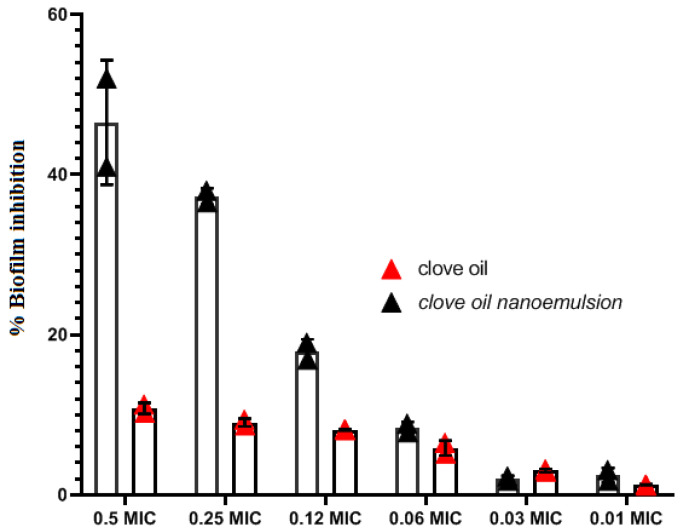
*S. aureus* biofilm inhibition in the presence of CL-emulsion and CL-nanoemulsion at Sub.MIC.

**Figure 4:**
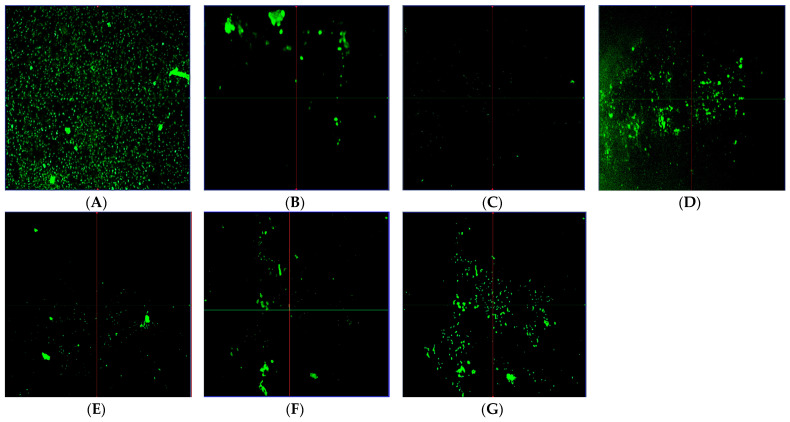
CLSM analysis of biofilms development by *S. aureus* without treatment (**A**) & biofilms development by *S. aureus* incubated with CL-nanoemulsion at 0.5 MIC, 0.25 MIC, and 0.06 MIC (**B**–**D**), respectively, for 24 h. Also, CLSM analysis of biofilms formed by *S. aureus* incubated with CL-emulsion at 0.5 MIC, 0.25 MIC, and 0.06 MIC (**E**–**G**), respectively, for 24 h.

Biofilm suppression was determined with a conventional crystal violet technique. At 0.5 × MIC and 0.25 × MIC, the antibiofilm efficacy of CL-emulsion and CL-nanoemulsion against *S. aureus* decreased the development of biofilm by 10.3 and 9.4% and 52 and 36.5%, respectively. Bacterial biofilm inhibition of CL-emulsion and CL-nanoemulsion showed considerable biofilm-inhibiting action against *S. aureus* (*p* < 0.05) (Figure 3). This medication had the strongest inhibitory capacity for biofilm formation against *S. aureus* at tested sub-MIC dosages without hindering planktonic development. The granularity of CL-nanoemulsions constitutes one of the key factors in their influence on biofilm inhibition action since tiny particles have a greater surface area for contact with microbes [44,45]. The presence of the *S. aureus* biofilm generated on the coverslip glass was visualized using CLSM. The microscopy image was obtained 48 h following the CL-nanoemulsion treatment at 0.5 MIC. Qualitative assessment to confirm biofilm inhibition was generated using Live/Dead labeling in CLSM (Figure 4). The untreated biofilm had numerous live adhering cells that had been well incorporated into the biofilm (Figure 4A). Biofilm treated with CL-nanoemulsion at 0.5, 0.25, and 0.06 MIC showed a small number of dead cells among the population (Figure 4B–D). Biofilm treated with CL-emulsion at 0.5, 0.25, and 0.06 MIC showed a small number of dead cells among the population (Figure 4E–G). When produced in the absence of the CL-nanoemulsion, dense biofilm forms with a tight topology characterized by a big cluster shape, and uniformly dispersed fluorescent green colors of active cells can be readily seen in the control biofilm. In contrast, the alleged CL-nanoemulsion might be an effective instrument for disrupting the biofilm and preventing its adhesion to the outer layer of the coverslips while also preventing the evolution of *S. aureus*. This finding matched up with our investigations on crystal violet biofilm inhibition. The susceptibility of mature biofilm toward CL-nanoemulsion was also seen with a substantial decrease (*p* < 0.01) in bacterial counts in biofilms treated in comparison to control biofilm. Our results agree with Chaieb et al. [46], who showed that *P. aeruginosa* strain creation of biofilm resulted in biofilm breakdown, with biofilm losing its rigidity entirely in the absence of living cells. This demonstrates the excellent effectiveness of levofloxacin loaded with CL-nanoemulsion in the elimination of preformed biofilms.

### 2.6. Antifungal Activity

Antifungal activity of CL-emulsion and CL-nanoemulsion was assessed against *C. albicans*, *C. neoformans*, *A. brasiliensis*, *A. flavus*, and *A. fumigatus*, where both MIC and MFC were determined as shown in Table 2. Results revealed that CL-nanoemulsion exhibited antifungal activity higher than CL-emulsion toward all selected fungal strains. Moreover, MICs of CL-nanoemulsion were 12.5, 3.12, 0.78, 1.56, and 1.56 mg/mL against *C. albicans*, *C. neoformans*, *A. brasiliensis*, *A. flavus*, and *A. fumigatus*, respectively. Also, MFCs of CL-nanoemulsion were 12.5, 6.25, 3.12, 1.56, and 6.25 mg/mL, respectively. These findings demonstrate the potential of CL-nanoemulsion against all investigated fungus strains, where the most sensitive strains were *A. brasiliensis* and *A. flavus*, but *C. albicans* was the least sensitive strain, among others. On the other hand, CL-emulsion showed weak antifungal activity toward all investigated fungal strains, where MICs were 50, 25, 6.25, 6.25, and 12.5 mg/mL against *C. albicans*, *C. neoformans*, *A. brasiliensis*, *A. flavus*, and *A. fumigatus*, respectively. Moreover, MFCs were 100, 25, 25, 12.5, and 50 mg/mL, respectively. Alghaith et al. [47] found that CL-nanoemulsion has antifungal activity toward the dermatophyte fungus *Trichophyton rubrum*. Another study reported that CL-nanoemulsion has promising antifungal activity against *A. niger* ATCC 1015 and *C. albicans* ATCC 3153 in which the impediment areas were 2.13 and 3.19 mm, respectively. Antifungal activity of CL-nanoemulsion may be attributed to the presence of eugenol, which can disrupt fungal cell membranes or inhibit germination and sporulation of the fungus. Also, it may be attributed to CL-nanoemulsion having the ability to inhibit ergosterol synthesis, inhibit enzymes of cell wall synthesis, altering of the morphology of the cell wall, and produce ROS [48].

### 2.7. Cytotoxic Effect of CL-Emulsion and CL-Nanoemulsion

**Figure 5:**
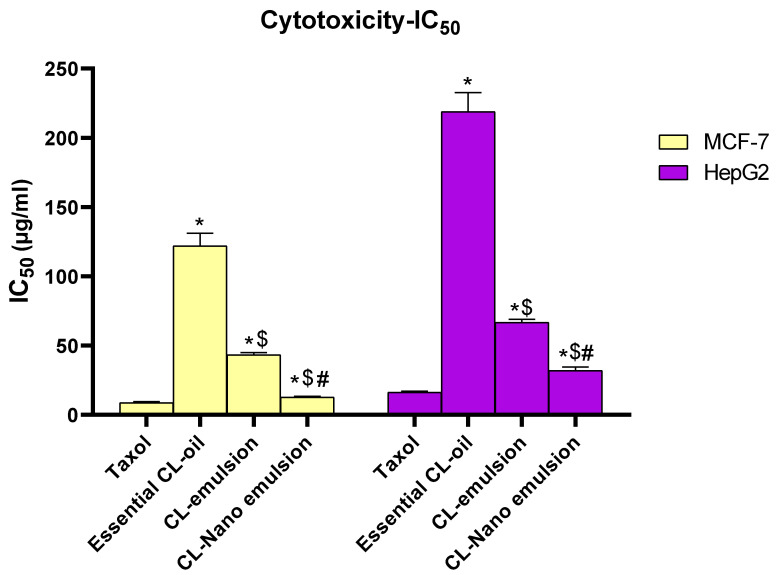
Cytotoxic effect of Taxol, CL-emulsion, and CL-nanoemulsion toward MCF-7 and HepG2 cell lines. The results are presented as means ± SD from three different tests, with * denoting a significant *p* value from the Taxol group at *p* < 0.001, $ denoting a significant *p* value from the essential CL-oil group at *p* < 0.001, and # indicating a significant *p* value from the CL-emulsion group at *p* < 0.001.

Nanoemulsions are employed as drug carriers to deliver medicines and phytochemicals to cells in an efficient way. They enhance the biological effects of their ingredients, including their antibacterial, antioxidant, and anticancer capabilities. The use of nanoemulsions as secure, biocompatible, and effective drug delivery devices has significantly changed cancer treatment plans and demonstrated a high level of safety [49]. Numerous research has used thyme, eucalyptus, cinnamon, and clove oil essential oils as therapeutic nanoemulsions [50]. In the current study, essential CL-oil (dissolved in DMSO), CL-emulsion, and CL-nanoemulsion were tested for their cytotoxic effects on HepG2 liver cancer cells and MCF-7 breast cancer cells, respectively. The most potent cytotoxic effect was seen in MCF-7 cells, as shown by the lowest IC50 values (Figure 5). Treatment with CL-nanoemulsion produced the lowest IC50 value of 12.93 ± 0.49 µg/mL. Additionally, treatment with CL-emulsion resulted in IC50 values of 43.36 ± 1.63 µg/mL, while the IC50 for Taxol was 8.90 ± 0.73 µg/mL.

### 2.8. Effect of CL-Emulsion and CL-Nanoemulsion on CASP8 and CASP9 Activities

**Figure 6:**
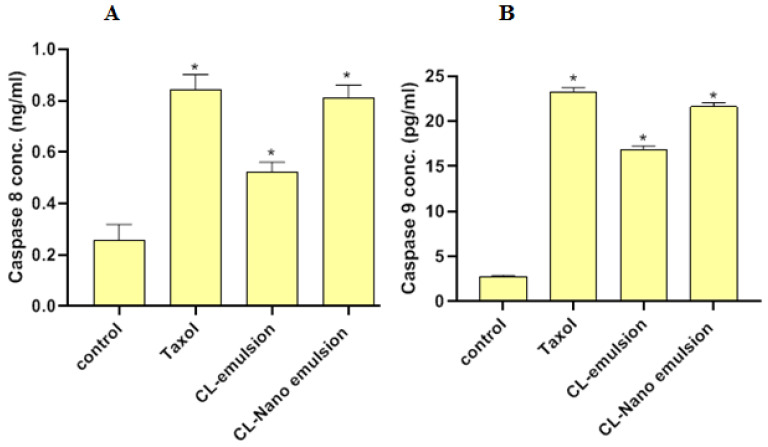
Effects of Taxol, CL-emulsion, and CL-nanoemulsion on CASP8 and CASP9 ((**A**,**B**), respectively) in MCF-7 cells compared to Taxol. The data are shown as mean ± SD from three separate tests, with * indicating a significant *p* value from the control group at *p* < 0.001.

In Figure 6, we see how CL-emulsion and CL-nanoemulsion affect the apoptotic markers CASP8 and -9. Treatment of MCF-7 cells with CL-emulsion substantially boosted CASP8 and CASP9 activity (0.523 ± 0.037 ng/mL and 16.9 ± 0.38 pg/mL, respectively) compared to the control (0.257 ± 0.061 ng/mL and 2.714 ± 0.19 pg/mL, respectively). In addition, compared to CL-emulsion treatments, CASP8 and -9 activities were shown to be greatest after being exposed to CL-nanoemulsion (0.811 ± 0.049 ng/mL and 21.63 ± 0.42 pg/mL, respectively). Apoptosis activates DNA fragmentation enzymes through CASP8 and -9 activations [51]. These compounds caused MCF-7 cells to undergo apoptosis via CASP8 and -9 activations.

### 2.9. Effect of CL-Emulsion and CL-Nanoemulsion on VEGFR-2

**Figure 7:**
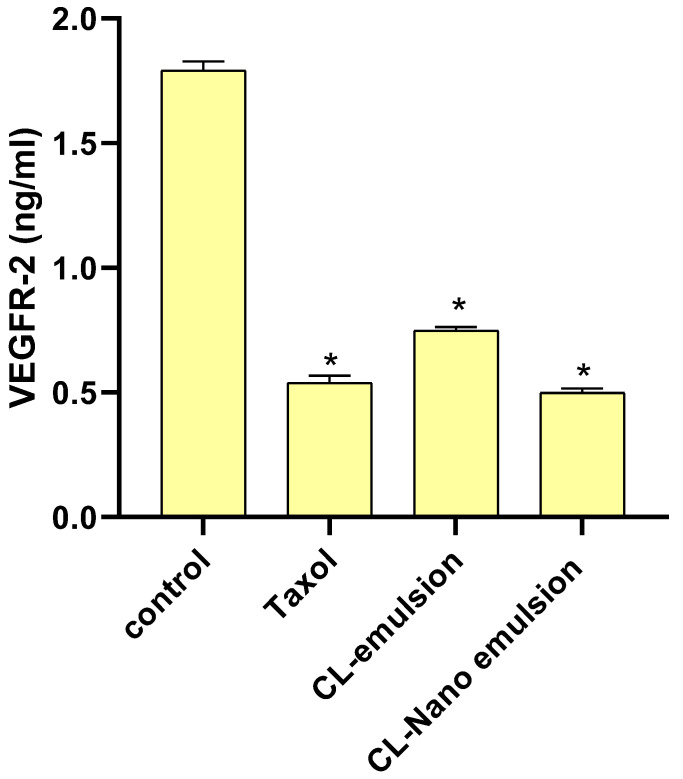
Effects of Taxol, CL-emulsion, and CL-nanoemulsion on VEGFR-2 in MCF-7 cells compared to Taxol. Statistics are presented as mean ± SD from three separate tests, with * indicating a significant *p* value from the control group at *p* < 0.001.

Figure 7 shows that the levels of VEGFR-2 were dramatically reduced by CL-emulsion and CL-nanoemulsion compared to the control (1.793 ± 0.036 ng/mL).

When comparing CL-emulsion (0.748 ± 0.016 ng/mL) to CL-nanoemulsion (0.499 ± 0.017 ng/mL), VEGFR-2 levels were found to be much lower in the latter. Overactivation of VEGFR-2 is known to drive angiogenesis that promotes solid tumor development, which corroborated the findings of Falcon et al. For many cancers, including breast cancer, blocking the VEGFR-2 pathway has emerged as an essential therapeutic strategy [52,53,54].

The dependability of cellular proliferation and development can be attributed to the role of apoptosis pathways, which are impaired in cancer cells. These cancer cells inhibit apoptotic pathways, leading to a reduction in apoptotic gene expressions and ultimately preventing apoptotic death. Caspases 8 and 9 are recognized as the effectors of the apoptosis response, and their primary function is to facilitate the induction of the tumor cells’ death. The apoptotic effect on breast cancer cells has been found to be upregulated by the cytotoxic properties of several plant essential oils, including Syzygium aromaticum (clove) [55]. Clove is noted for its significant antioxidant properties attributed to its constituent compounds such as tannins, flavonoids, glycosides, and volatile phenolic oils such as eugenol and acetyl eugenol. The phytochemical ingredients present in cloves exhibit potent antioxidant, antiproliferative, antimicrobial, disinfectant, and anti-inflammatory properties, thereby rendering them suitable agents for cancer chemoprevention [56]. The efficacy of this compound can be further enhanced by using a nanoemulsion formulation. Nanoemulsions have garnered significant attention from scientists in the pharmaceutical industry and in the discipline of cancer treatment due to their capacity to enhance the solubility and efficacy of therapeutic agents, including tetanus toxoid, insulin, and anticancer agents, during drug delivery [57].

In addition, it is noteworthy that plant-derived bioactive substances, such as phenolic and flavonoids, exhibit therapeutic potential but possess limited solubility in polar solvents. Consequently, the efficacy of plant-based anticancer substances is hindered by their inadequate solubility and gastrointestinal absorption. The enhancement of solubility and gastrointestinal uptake of plant bioactive substances can be achieved by incorporating them into a suitable carrier, including nanoemulsions. This approach can effectively address the aforementioned issue. The utilization of nanoemulsions in drug delivery can be advantageous due to the enhanced solubility of the therapeutic agent, the prolonged half-life, and the potential to surmount the resistance of cancerous cells toward chemotherapy [58].

## 3. Material and Methods

### 3.1. Chemicals and Reagents

Taxol (paclitaxel) was bought from Sigma Chemical in St. Louis, MO. Sigma Aldrich (Sigma, St. Louis, MO, USA) supplied the 3-(4,5-Dimethyl-2-thiazolyl)-2,5-diphenyl-2H-tetrazolium bromide (MTT) and the dimethyl sulfoxide (DMSO). From Gibco (Gibco, TFS frpm PPA, Pasching, Austria) we obtained fetal bovine serum (FBS), phosphate buffer saline (PBS), Dulbecco’s modified Eagle’s medium (DMEM), penicillin/streptomycin (Pen/Strep) solution, and trypsin-EDTA. Phosphotungstic acid, ethanol 95%, SYTO 9, propidium iodide (PI), Tween 80, and resazurin dye were purchased from Sigma Aldrich, Sparks, NV, USA.

### 3.2. Preparation of CL-Nanoemulsion and CL-Emulsion

The steam distillation method was used for the extraction of clove essential oil according to the method used by [59]. Dried and ground clove flower buds (50 g) were put in a steam flask. The steam distillation lasted 6 h. The recovered condensate was distilled again using n-hexane as the solvent. By evaporating the n-hexane, clove oil was produced. To prepare CL-nanoemulsion, 5 mL of Tween 80 was added slowly to 20 mL of CL with gentle stirring for 40 min, and then completed to 100 mL with distilled water. The mixture was sonicated using an ultrasonicator for 40 min at 350 W. The essential oil emulsion was made in the manner described above but without the use of a sonicator.

### 3.3. Measurement of CL-Nanoemulsion Droplet Size

At room temperature, the Zeta Nano ZS (Malvern Instruments, UK) was used to measure the size of the CL-nanoemulsion droplets using a dynamic light scattering analysis. Before testing, 30 µL of CL-nanoemulsion was diluted with 3 mL of water at 25 °C. The mean of the Z-average of three separate batches of the CL-nanoemulsion was used to express particle size information. We examined the CL-nanoemulsion’s droplet size and polydisparity index (PDI).

### 3.4. Transmission Electron Microscopy (TEM)

On a film-coated 200-mesh copper specimen grid, 20 µL of sample was placed. Then, one drop of 3% phosphotungstic acid was used to stain the grid and allowed to dry for 3 min. Using a TEM microscope (Tecnai G20, Super twin, double tilt, FEI, The Netherlands) set to 200 kV, the coated grid was analyzed [60]. TEM of CL-nanoemulsion was carried out at RCMB, Al-Azhar University, while TEM of CL-emulsion was carried out at the Agricultural Research Center (ARC), Egypt.

## 4. Antibacterial Activity

### 4.1. Microorganisms

*Bacillus cereus* ATTC 11778, *Staphylococcus aureus* ATCC 25923, *Escherichia coli* ATCC 35218, and *Klebsiella oxytoca* ATCC 51983 were selected for antibacterial screening, while *Staphylococcus aureus* (MSSA) ATCC-25923 was used as positive controls of biofilm production.

### 4.2. MIC Determination by Resazurin Dye Method

Microbroth dilution method by resazurin dye method was applied for the determination of antimicrobial activities [61]. First, 100 µL of bacterial suspension was added to the nutrient broth and cultured for 24 h at 37 ± 2 °C to test the antibacterial effectiveness [62,63,64,65]. Microdilutions of overnight established culture strains were grown in Luria-Bertani broth in 96-well plates (McFarland turbidity of 0.5). CL-emulsion and CL-nanoemulsion at various concentrations (20, 10, 5, 2.5,1.25, 0.62, 0.31, 0.15, and 0.07%) were added [66]. The lowest concentration of chemicals tested at which the dye colour changed. The minimum bactericidal concentration (MBC) was determined when no colonies formed on the agar plate.

### 4.3. Evaluation of Antibiofilm Activity

The effect of CL-emulsion and CL-nanoemulsion on *Staphylococcus aureus* (MSSA) ATCC-25923 biofilm development was determined by the crystal violet staining technique [67,68]. Six levels lowered from 0.5 × MIC of CL-emulsion and CL-nanoemulsion were added to 96-well plates as previously described. For 48 h, the liquid combination was eliminated, and the wells were stained for 15 min with 0.1 mL 0.4% crystal violet after being cleaned twice with sterile water. Following that, the dye attached to the biofilm was dissolved using 95% ethanol. The plates were read at 492 nm in an ELISA reader, and all tests were done in triplicate [46,69]:

### 4.4. Observation of Biofilm Reduction by Confocal Laser Scanning Microscopy (CLSM)

At 0.5 MIC, one-milliliter cell suspensions of *Staphylococcus aureus* ATCC 25923 were distributed onto microtiter plate wells containing CL-emulsion and CL-nanoemulsion. Following incubation, the coverslips were carefully rinsed with 0.01 M buffered saline to carefully eliminate non-adhered bacteria and were stained with 500 µL of a combined dye solution of SYTO 9 and propidium iodide (PI) [44]. Following that, visualization was carried out using a ZEISS LSM laser scanning microscope with a 40 oil-immersion lens with settings for the green signal of 488 nm and the red signal of 461 nm and long-pass emission filters of 500–550 nm and 590–650 nm, respectively [70].

### 4.5. Antifungal Activity

Antifungal activity of CL-emulsion and CL-nanoemulsion was assessed against *Candida albicans* ATCC 90028, *Cryptococcus neoformans* ATCC 14116, *Aspergillus brasiliensis* ATCC 16404, *A. flavus* RCMB 02782, and *A. fumigatus* ATCC 204305. The MIC and MFC of the CL-emulsion and CL-nanoemulsion were analyzed using the broth microdilution method [71]. The microdilution broth method was applied for detecting the MIC of all tested fungal stains. Briefly, in a microplate, 10 µL of each fungal strain was added to Sabouraud Dextrose broth amended with different concentrations of CL-emulsion and CL-nanoemulsion (100–0.19 mg/mL), and then incubated at 30 °C for 48 h. For detection of MIC for unicellular fungi, 20 µL of resazurin dye was added. A visual assessment was done from blue to pink dye inside viable cells. On the other hand, MIC for filamentous fungi was detected by examining growth visually without adding dye [7,8,72]. To detect minimum fungicidal concentration (MFC), 100 µL was transferred from a well that had no visible growth to Sabouraud Dextrose plates and then incubated at 30 °C for 48 h.

## 5. Anticancer Activity

### 5.1. Cell Lines

Cell lines for both breast (MCF-7) and liver (HepG2) cancers were obtained from ATCC (Manassas, VA, USA) and grown in DMEM (Invitrogen, Carlsbad, CA, USA) with 10% fetal bovine serum (FBS) and 1% pen/strep solution (TFS Inc., City of Fairfax, VA, USA) at 37 °C (with 5% carbon dioxide).

### 5.2. Cell Viability Assay

The MTT assay was used to quantify cytotoxic activity [73]. In 96-well plates, the cells were planted at a density of 1.2 × 10^4^ cells/well and given 24 h to grow. After 24 h, the media with the various essential CL-oil (dissolved in DMSO), CL-emulsion, and CL-nanoemulsion concentrations were changed. After 48 h, the MTT test was carried out by adding 100 μL (5 mg/mL of MTT in PBS) followed by 4 h of incubation at 37 °C in the wells. Each well had 100 μL of DMSO added to it to dissolve the formazan crystals. Ten minutes were spent incubating the plates at 37 °C. Microplate reader readings at 570 nm were used to calculate optical densities [74].

### 5.3. Assessment of Caspase-8 (CASP8) and Caspase-9 (CASP9) Activities and VEGFR-2

ELISA kits from DRG International Inc. (Springfield, NJ, USA) were used to test CASP8 (human, EIA-4863) and CASP9 (human, EIA-4860). VEGFR-2 was measured using an ELISA kit (Catalogue #: OKAG02083) (AVIVA system biology, San Diego, CA, USA) per manufacturer instructions.

### 5.4. Statistical Analysis

GraphPad Prism 8.0 examined all findings. Means ± SD were reported for at least three separate experiments (*n* = 3). ANOVA and Tukey’s multiple comparisons tests examined all groups’ significant differences. *p* < 0.05 was significant.

## 6. Conclusions

In the current study, CL-nanoemulsion and CL-emulsion were successfully prepared using an ecofriendly method. Also, CL-nanoemulsion and CL-emulsion were characterized using DLS and TEM. Results confirmed that the prepared CL-nanoemulsion was in nano form with a spherical shape. Results revealed that CL-nanoemulsion exhibited outstanding antibacterial activity compared with CL-emulsion, where CL-nanoemulsion possesses the lowest MIC ranging from 0.31 to 5 mg/mL toward pathogenic Gram-negative and Gram-positive bacteria. Moreover, the antibiofilm efficacy of CL-nanoemulsion against *S. aureus* has significantly decreased the development of biofilm compared with CL-emulsion. Furthermore, CL-nanoemulsion displayed promising antifungal action against both unicellular and multicellular fungi. Also, the anticancer impact of CL-nanoemulsion is due to the induction of apoptosis in MCF-7 breast cancer cells by increasing caspase-8 and -9 activity and decreasing VEGFR-2 compared with CL-emulsion.

## Figures and Tables

**Table 1:** MIC (mg/mL) and MBC (mg/mL) of CL-emulsion and CL-nanoemulsion.

	*B. subtilis*		*S. aureus*		*E. coli*		*K. oxytoca*	
	MIC (mg/mL)	MBC (mg/mL)	MIC	MBC	MIC	MBC	MIC	MBC
CL-emulsion	1.25	2.5	2.5	5	10	20	5	10
CL-nanoemulsion	0.31	0.62	0.62	1.25	1.25	2.5	5	10
Ciprofloxacin	0.62	1.25	0.62	1.25	1.25	1.25	0.62	1.25

**Table 2:** MIC (mg/mL) and MFC (mg/mL) of CL-emulsion and CL-nanoemulsion toward fungal strains.

Fungal Strains	CL-Emulsion		CL-Nanoemulsion		Voriconazole	
	MIC *	MFC	MIC	MFC	MIC	MFC
*C. albicans*	50	100	12.5	12.5	25	50
*C. neoformans*	25	25	3.12	6.25	12.5	12.5
*A. brasiliensis*	6.25	25	0.78	3.12	6.25	12.5
*A. flavus*	6.25	12.5	1.56	1.56	3.12	3.12
*A. fumigatus*	12.5	50	1.56	6.25	6.25	25

* MIC means minimum inhibitory concentration (mg/mL).

## Data Availability

All data and materials are viable.
