# Correction: Shehabeldine et al. Antimicrobial, Antibiofilm, and Anticancer Activities of *Syzygium aromaticum* Essential Oil Nanoemulsion. *Molecules* 2023, *28*, 5812

**DOI:** 10.3390/molecules29235574

**Published:** 2024-11-26

**Authors:** Amr M. Shehabeldine, Ahmed S. Doghish, Walaa A. El-Dakroury, Mahmoud M. H. Hassanin, Abdulaziz A. Al-Askar, Hamada AbdElgawad, Amr H. Hashem

**Affiliations:** 1 Botany and Microbiology Department, Faculty of Science, Al-Azhar University, Nasr City 11884, Egypt; 2 Department of Biochemistry, Faculty of Pharmacy, Badr University in Cairo (BUC), Badr City 11829, Egypt; 3 Biochemistry and Molecular Biology Department, Faculty of Pharmacy (Boys), Al-Azhar University, Nasr City 11231, Egypt; 4 Department of Pharmaceutics and Industrial Pharmacy, Faculty of Pharmacy, Badr University in Cairo (BUC), Badr City 11829, Egypt; walaa.ahmed2@buc.edu.eg; 5 Ornamental, Medicinal and Aromatic Plant Disease Department, Plant Pathology Research Institute, Agricultural Research Center (ARC), Giza 12619, Egypt; dr.hassanin.1978@gmail.com; 6 Department of Botany and Microbiology, Faculty of Science, King Saud University, P.O. Box 2455, Riyadh 11451, Saudi Arabia; aalaskara@ksu.edu.sa; 7 Integrated Molecular Plant Physiology Research (IMPRES), Department of Biology, University of Antwerp, 2022 Antwerp, Belgium; hamada.abdelgawad@uantwerpen.be

## Error in Figure

In the original publication [1], there was a mistake in Figure 4 (C, E, F and G). The inclusion of these images was an unintended error. During the time of article preparation, there existed another accurate figure, but due to a mistake, the old, published figure was inadvertently used. Unfortunately, this oversight went unnoticed by the senior author. The corrected Figure 4 appears below. The authors state that the scientific conclusions are unaffected. This correction was approved by the Academic Editor. The original publication has also been updated.

## Figures and Tables

**Figure 4:**
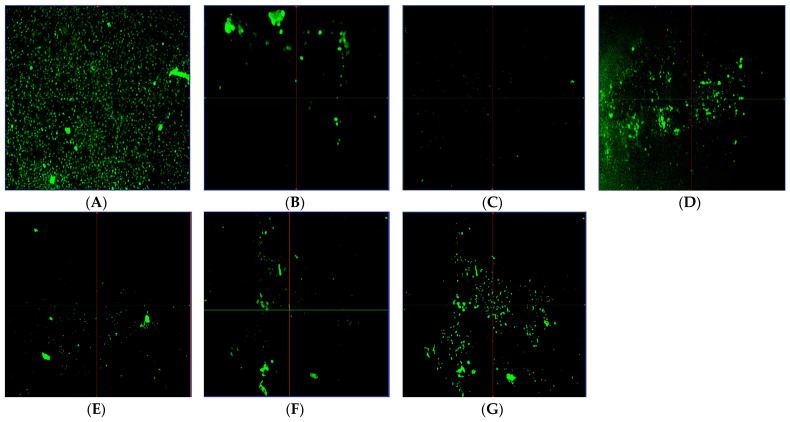
CLSM analysis of biofilms development by *S. aureus* without treatment (**A**) & biofilms development by *S. aureus* incubated with CL-nanoemulsion at 0.5 MIC, 0.25 MIC, and 0.06 MIC (**B**–**D**), respectively, for 24 h. Also, CLSM analysis of biofilms formed by *S. aureus* incubated with CL-emulsion at 0.5 MIC, 0.25 MIC, and 0.06 MIC (**E**–**G**), respectively, for 24 h.
